# Ageing versus developmental silencing: Answers from the epigenome

**DOI:** 10.1111/febs.70221

**Published:** 2025-08-12

**Authors:** Kirsten C. Sadler, Mekayla A. Storer, N. Sumru Bayin

**Affiliations:** ^1^ Program in Biology and Center for Genomics and Systems Biology NYU Abu Dhabi UAE; ^2^ Cambridge Stem Cell Institute University of Cambridge UK; ^3^ Department of Physiology, Development and Neuroscience Cambridge University UK; ^4^ Gurdon Institute Cambridge University UK

**Keywords:** ageing, development, epigenetics, epigenomics, regeneration

## Abstract

A strong regenerative capacity is a hallmark of youth. From the tadpole's tail to the mammalian brain, young animals of many species can repair or regrow damaged tissues more effectively than older animals. Here, we take a broad perspective on ageing, inclusive of the transition from the developmental processes of embryogenesis through maturation to adulthood, as well as the processes that occur as an animal reaches the end of its lifespan. In some cases, the loss of regenerative capacity occurs once development is complete, and in others it occurs in the latter part of the animal's life. Regardless, the loss of regenerative capacity is caused by a failure to activate genes required for successful regeneration. This, in part, can be attributed to restructuring of the epigenome.

## Graphical abstract

It is easier for young animals to regenerate damaged or missing tissues. In this Viewpoint, we propose that this is, in part, attributed to epigenetic changes, with chromatin becoming more closed and inaccessible as animals age. The timing of when this occurs differs depending on the tissue—some during the late developmental stages and others at the end of life.

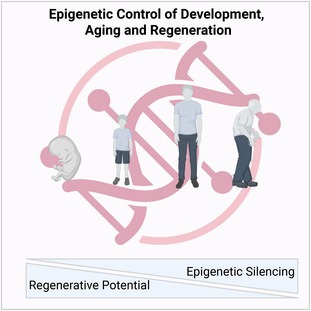

**Abbreviations**
DNA: deoxyribonucleic acid
H3K27me3: histone 3 lysine 27 trimethylation
H3K9me2: histone 3 lysine 9 dimethylation
H3K9me3: histone 3 lysine 9 trimethylation
RNA: ribonucleic acid
TSSs: transcription start sites

## Introduction

**Fig. 1:**
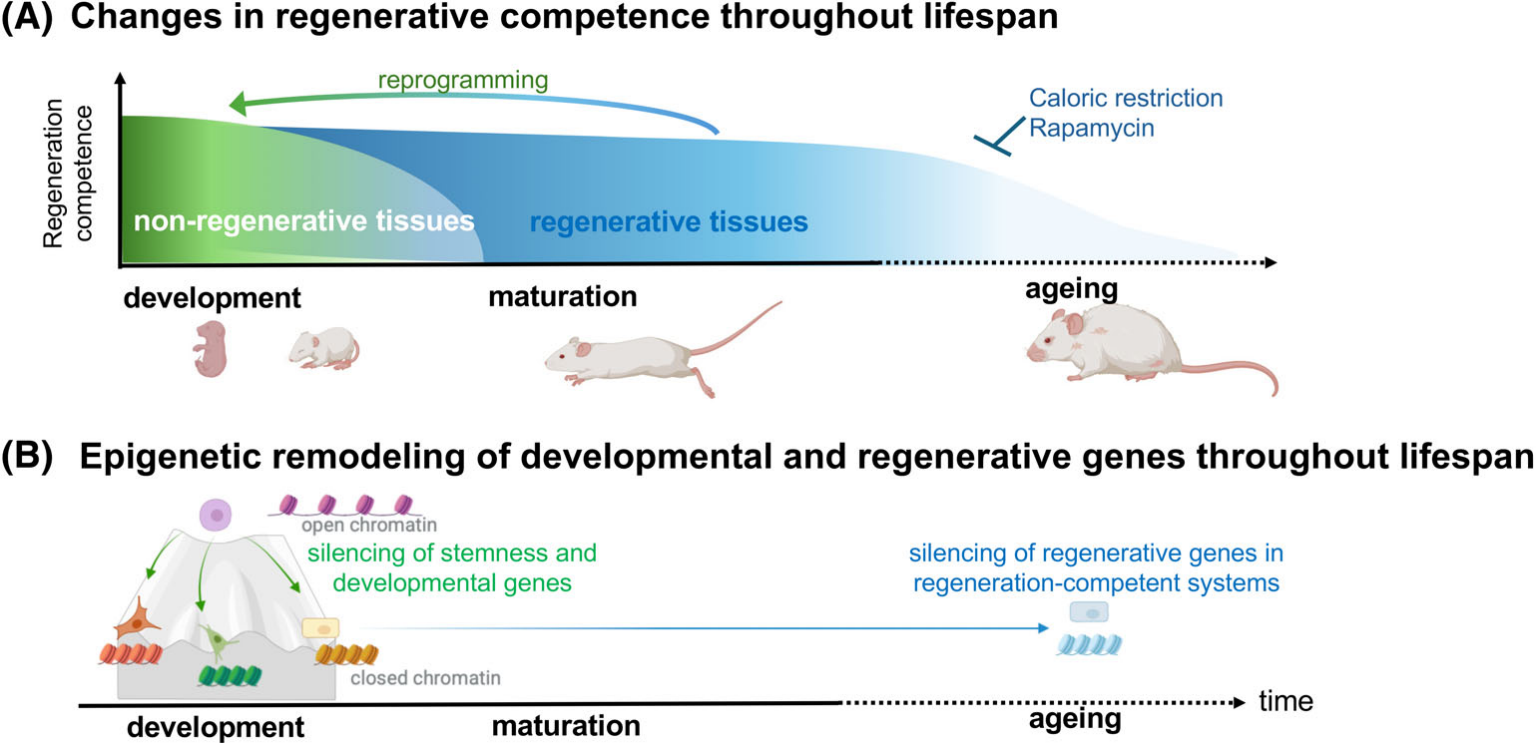
Epigenetic changes over the lifespan can restrict regenerative potential. (A) Some mammalian tissues lose regenerative capacity during the process of development and maturation, while it declines in others towards the end of the lifespan. (B) The shift from open chromatin to closed heterochromatin dictates the expression of genes that regulate cell identity and occurs during development. Epigenetic processes that occur during ageing are also proposed to silence pro‐regenerative genes in those tissues that maintain regeneration competence in adulthood. This figure was generated, in part, using BioRender.

During early development as cell transition from multipotency to committed cell fates, the chromatin remains accessible so that cells can respond to differentiation cues and activate genes required for differentiation and acquisition of identity. As the organism matures, chromatin closes around genes required for plasticity as well as those that play important roles in regeneration, sequestering key cell cycle drivers and repair programmes in inaccessible heterochromatin. While this has benefits for the maintenance of cell identities that carry out complex physiologies required by adult animals, the consequence in some species is that this chromatin shift limits regenerative potential (Fig. 1A).

Developmental programmes that enable regeneration are likely retained in life‐long regenerators, such as planarians, axolotls and zebrafish. With a few exceptions, such as the amazingly regenerative spiny mouse [1, 2], mammals stand out as a notable exception, as most tissues that can regenerate in juveniles or young adults lose this ability during maturation and ageing [3] (Fig. 1B). However, a few tissues—such as the liver [4] and digit tips [5]—retain their regenerative capacity into adulthood, yet even in these tissues, regenerative capacity declines with ageing [6, 7, 8]. A major goal of regenerative medicine is to improve regenerative capacity. This could be achieved by preventing oxidative stress, DNA damage and inflammation that are induced by injury, and additionally by reversing the metabolic dysfunction and epigenetic changes that occur during ageing [9, 10, 11, 12].

To understand what promotes regeneration and what prevents it, researchers study organisms that can regenerate throughout life. Additionally, many focus on the few mammalian tissues that retain regenerative ability in adulthood. Other approaches are to study tissues that do not naturally regenerate—such as the mammalian brain, limbs or heart—to identify the factors that limit regeneration and to develop strategies to block these factors and thereby promote regrowth. Results from both approaches have converged on the understanding that a key difference between regeneration‐competent and regeneration‐incompetent cells is whether pro‐regenerative signals can access and activate gene targets required for regeneration. This places epigenetic control at the centre of regenerative biology [13].

Epigenetic repatterning during development, maturation and ageing regulates cell identity and proliferative capacity, and this repatterning can also explain the changes in regenerative capacity. It is now understood that in embryos, signals that are present as early as the zygote and continue through developmental time set an epigenetic pattern that dictates when and where genes dictating cell identity and genes required for proliferation are activated [14, 15, 16]. Whether epigenetic changes are causal or correlative during ageing remains poorly understood. It is possible that the erosion of epigenetic patterns occurs as a consequence of DNA damage repair, accumulated errors in DNA replication or environmental influences that limit the building blocks required for epigenetic modifications [11].

A unifying concept is that regenerative‐competent mammalian tissues maintain a pro‐regenerative epigenetic code [17], while nonregenerative tissues sequester these genes in heterochromatin. We propose that a similar paradigm governs the regenerative potential that changes during the lifespan. Importantly, even those tissues that retain regenerative capacity in adults undergo epigenetic changes during ageing [10, 11, 18] and these changes can silence pro‐regenerative genes. Conversely, discoveries that differentiated cells can be reprogrammed to pluripotent states indicate that the epigenetic marks required for cell identity and function are not indelibly fixed on the genome. An excellent recent review proposes a mode in which most regenerative processes proceed through a series of steps, and one way to identify why regeneration does not work in some tissues is to identify which of the steps are defective [19]. One possibility is that the genes required at each stage of regeneration are regulated by distinct chromatin modifications, or that different regenerative contexts have evolved unique epigenetic signatures to control similar biological processes.

We present examples of regeneration capacity loss as part of developmental maturation in the mammalian brain and heart and the tadpole limb, and discuss the loss of regenerative capacity in the ageing liver. While specific molecular players that influence epigenetic marks may vary across tissues, the overarching principle remains the same: the epigenome dictates what a cell can become, how it functions and whether it can regenerate.

## Epigenetic silencing: Development vs. ageing

Epigenetic silencing involves packaging regions of the genome into heterochromatin, either facultative or constitutive. Facultative heterochromatin can quickly transition to a less condensed, more accessible euchromatin state, while constitutive heterochromatin is more stably repressed and resistant to change. Constitutive heterochromatin is associated with canonical heterochromatin marks, such as histone 3 lysine 9 tri‐or di‐methylation (H3K9me3 or H3K9me2) or DNA methylation and is also regionally segregated, with silenced regions of the genome localized to the nuclear lamina or in heterochromatin foci, preventing transcription factor access [20, 21].

In facultative heterochromatin, genes are marked with both activating and repressive epigenetic marks, with H3K27me3 emerging as an important mark that regulates poised genes [22]. Such poised chromatin is open and permissive—allowing transcription factor binding, enhancer‐promoter looping and RNA polymerase loading—yet the genes remain transcriptionally silent due to the presence of the repressive H3K27me3 mark. These genes are accessible, and when the appropriate stimulus triggers their activation, H3K27me3 and other repressive marks are removed, and activating marks are deposited, promoting transcription. H3K27me3 is central to both developmental and adult regenerative gene programmes; however, a distinction between these processes is that DNA methylation patterns are eroded during ageing but are relatively unchanged during cell differentiation and tissue maturation.

During development, it is well established that genes essential for pluripotency become epigenetically silenced, while genes required for cell identity are shifted to euchromatin and are either poised or actively transcribed [22]. As development proceeds and stem cells differentiate into specialized cells, genes required for plasticity become segregated into heterochromatin, while those that maintain cell identity are positioned in accessible euchromatin [21]. This is the basic principle of the field studying the chromatin basis of cell identity and is a critical aspect of understanding cellular reprogramming (Fig. 1B).

In contrast to developmental epigenetic changes which are regulated and stereotypical, the epigenetic changes that occur during ageing are more haphazard [11, 23, 24, 25]. Changes in the aged epigenome result in disordering of the DNA methylome and an unmasking of the intergenic regions. This results in the activation of transposable elements [26, 27], which then contribute to DNA damage and the subsequent activation of the DNA damage response—key mechanisms driving age‐related declines in cellular function and regenerative capacity. DNA methylation is the best studied of the age‐related epigenetic changes, and one hypothesis is that errors of methylation maintenance during DNA replication accumulate with age; this can contribute to methylation erosion [28]. Although epigenetic remodelling during ageing differs from that occurring in development, both processes likely influence the activation of genes essential for regeneration.

## Development to maturation: Silencing and its effect on regenerative capacity

The developing epigenome is regeneration permissive, allowing progenitors to respond rapidly to injury‐induced signals and activate proliferation and differentiation programmes. The advantage to silencing these programmes after maturation likely prevents aberrant reprogramming and tumorigenesis in adulthood [29]. Importantly, the cellular environment in adulthood shifts towards maintaining homeostasis rather than growth, and interventions that perturb this—such as chronic inflammation or injury to highly regenerative tissues, such as liver or the intestinal epithelium—can lead to neoplastic transformation. On the contrary, all highly regenerative animals do not have the same propensity to develop tumours, indicating that lots of regeneration does not directly correlate with lots of cancer, and the interplay between regenerative signals and those that drive tumours requires more study.

While epigenetic remodelling is most prominent as developmental processes wind down and cell identity is solidified, there are several tissues that also display these changes during maturation in postnatal and juvenile stages. The mammalian central nervous system is a good example of how regeneration competence is tightly regulated by developmental timing and maturation. Neonatal mice exhibit some regenerative capacity, but adults have virtually no ability to regenerate neurons following injury [30], as most neural progenitors are depleted in postnatal animals. The few progenitors that remain in neurogenic niches can continue to make new neurons, albeit at a very limited rate. However, even this minimal proliferative and neurogenic potential declines during ageing [31].

The cerebellum, a hindbrain region important for motor control, is an ideal model to study the relationship between developmental maturation and regenerative capacity. In neonatal mice, cerebellar progenitors can repair injury either via re‐entering the cell cycle or adaptive reprogramming, a multistep process that involves proliferation and fate switch [30, 32, 33, 34]. However, regenerative competency is limited to around birth, and there is no regeneration following injury in the adult cerebellum, despite the presence of stem‐like cells [30]. Our preliminary work to address this using chromatin accessibility studies of neonatal cerebellar stem cells and injury responsive stem‐like cells from adult mice reveals that neurogenic and proliferative genes are in a more open state in neonates compared with adults, consistent with a model of epigenetic mediated silencing of regenerative capacity in the brain (Fig. 1B). Further investigation in this model can uncover the molecular mechanisms driving brain regeneration and inform studies in other systems where developmental maturation reduces regeneration competency.

The mammalian heart is another example of a shift in regenerative capacity during maturation. Neonatal mice can regenerate their hearts following cardiac injury; however, this capacity is lost as cardiomyocytes mature and withdraw from the cell cycle shortly after birth [35]. Interestingly, the loss of cardiac‐regenerative capacity during early postnatal life correlates with dramatic changes in the epigenetic landscape [35, 36, 37, 38]. For example, cardiomyocytes from neonates, but not adults, proliferate in response to Notch activation, and this corresponded to an accumulation of repressive histone marks at the promoters of Notch‐responsive effectors during maturation, preventing adult cardiomyocytes from re‐entering the cell cycle [39]. This suggests that regeneration in adult hearts is limited by repressive epigenetic marks on pro‐regenerative genes and provides the promise of engineering changes to these marks to promote cardiac regeneration [38].

## Epigenetic barriers in nonregenerative tissues

While much of the work on epigenetics and regeneration has focused on how to activate pro‐regeneration genes, a less explored but equally important area is understanding how regeneration programmes are silenced in regeneration‐incompetent tissues. For instance, in contrast to the highly regenerative limbs of axolotl, frog limb regeneration varies across developmental stages: amputations at early limb bud stages regenerate, while this capacity declines as frogs undergo metamorphosis so that amputation in late tadpoles and adult frogs only results in the formation of a spike‐like structure [40]. In early‐stage tadpoles, limb regeneration is driven by the formation of a blastema [41]—a mass of proliferative, undifferentiated cells that can reform the missing structures. The loss of regenerative capacity after metamorphosis is linked to a failure to reactivate sonic hedgehog [42], analogous to the block in activation of Notch‐responsive genes in the adult mammalian heart. Interestingly, transplanting progenitor cells from regeneration‐competent stages along with key signalling molecules to older, regeneration‐incompetent amputated limbs restores regeneration, with cells from both the donor and the host making up the newly formed limb [43]. The loss of regenerative limbs in older frogs could be attributed to epigenetic silencing of these genes, preventing blastema formation and instead forming a fibrotic scar or spike, and the rejuvenation of this process by young progenitors and signalling molecules suggests that the silencing process can be altered.

## The ageing epigenome and loss of regenerative potential

Widespread epigenetic changes, especially to the repressive epigenome, are a hallmark of ageing in mammals. The stereotypical changes to DNA methylation during mammalian ageing form the basis for the much‐celebrated epigenetic clocks that read out biological age as a counter to chronological age [25, 44]. At the basis of the ageing epigenome is the disordering of the DNA methylation pattern [11, 24, 25]. In young cells, DNA methylation occupies transposable elements and other regions of the noncoding genome to maintain their silencing and genome organization. This change is described as having increased entropy, or randomization, unleashing transposons and potentially contributing to DNA damage and the increased inflammation observed in ageing tissues, *that is* inflamm‐ageing [45].

In contrast to the brain and heart, the liver can regenerate in response to resection or damage in adult mammals [4]. However, as the liver ages, methylome entropy increases, several repressive histone marks are repatterned, and the regenerative capacity of the liver declines [6, 7, 9, 23]. At the same time, DNA replication defects in aged hepatocytes induce replication stress [46], which is linked to errors copying the epigenetic pattern from parent to daughter cells [28]. Interestingly, the hepatic age‐associated epigenetic patterns are prevented by caloric restriction and rapamycin treatment and, surprisingly, are rejuvenated when the liver is stimulated to regenerate by partial hepatectomy [18, 24].

We defined an epigenetic code that is associated with regenerative capacity in the liver whereby many of the genes required for cell cycle re‐entry are poised in the quiescent liver and are then activated during regeneration [17, 47]. We suggest a model in which this code keeps pro‐regenerative genes in a ‘ready‐set‐go’ state, allowing a rapid and coordinated response to mitogens and coordinated activation during regeneration. Age‐related epigenetic changes may disrupt this poised state, restricting the ability of cell cycle genes to become activated when there is a regenerative stimulus. Another epigenetic mechanism proposed to contribute to regenerative decline was suggested by a recent finding that age‐related chromatin changes can lead to transcription initiating within gene bodies, rather than at canonical transcription start sites (TSSs). This was associated with the presence of ‘fuzzy’ TSS‐like motifs in regions of the genes marked by features of poised enhancers [48], suggesting a link between age‐related epigenetic changes and aberrant gene expression. A third mechanism by which epigenetic changes can impair regenerative capacity is through de‐repression of transposable elements, which is a common feature of many aged tissues [49]. Since some transposable elements trigger viral sensors, their activation is proposed as a mechanism of inflamm‐ageing, which in turn can suppress regeneration. This is important, as human livers with markers of inflammation have a lower regenerative potential following surgery [50]. Thus, epigenetic changes during ageing can have both direct and indirect effects on the ability of the liver and possibly other tissues to regenerate.

## Conclusions and future directions

This is an exciting time in regenerative biology, as understanding regeneration across tissues and species holds great potential for improving human health. One of the principal questions to address definitively is whether the epigenetic changes that occur during ageing are a cause or consequence of the ageing process; and from this, linking these chromatin‐based changes to functional changes in regenerative capacity.

To advance regenerative medicine, it is important to define the pro‐regenerative genes that are reversibly repressed during homeostasis through facultative heterochromatin, but become permanently silenced in regeneration‐incompetent tissues via constitutive heterochromatin. Once these genes are identified, it will be essential to characterize the specific chromatin modifications that restrict their expression and to determine how these marks are selectively deposited at pro‐regenerative loci. This knowledge would provide a foundation for strategies aimed at reactivating these genes to restore regenerative capacity.

Additional open questions include: How is the epigenome reshaped during ageing, and can age‐associated epigenetic changes be reversed to restore regenerative potential? Insights into these questions are beginning to emerge from studies of induced pluripotency in differentiated cells (Fig. 1B). Animal models will be essential for determining which of these epigenetic changes contribute to age‐related phenotypes. Addressing these questions is critical for developing strategies that can safely and effectively remodel the epigenome to promote regeneration—while avoiding uncontrolled activation and the associated risk of cancer.

Life‐long caloric restriction, resveratrol treatment and modulation of insulin signalling are among the most effective interventions known to extend lifespan, and they appear to do so by preserving a youthful epigenome. This suggests that metabolic states and signalling pathways can interact with the epigenome in ways that delay ageing. However, these interventions lose efficacy when applied later in life, highlighting a critical gap in our understanding—while we know how to delay ageing, true rejuvenation remains elusive. As new tissue‐specific signalling molecules and metabolic requirements for regeneration are identified, alternative approaches to deliver pro‐regenerative signals to tissues that have lost regenerative capacity would be a major advance. For such strategies to succeed, however, target cells must be able to respond by establishing a chromatin landscape that permits regeneration. Future work aimed at epigenetically reactivating regenerative potential in aged tissues must also account for extrinsic, regeneration‐limiting factors—such as chronic inflammation and accumulated damage—in order to fully unlock the tissue's capacity for repair.

## Author contributions

NSB, MS and KCS conceived of the idea, and wrote and edited the manuscript.

## Conflict of interest

The authors declare no conflict of interest.
